# Body roundness index, thyroid hormones, and threshold effects in US adults: a cross-sectional study from NHANES

**DOI:** 10.3389/fnut.2025.1539022

**Published:** 2025-07-03

**Authors:** Sijia Yang, Kun Liao, Lu Zhou, Shengbo Zhang, Jianchao Wu

**Affiliations:** ^1^Department of Thyroid Diagnosis and Treatment Center, Zhuhai People’s Hospital (The Affiliated Hospital of Beijing Institute of Technology, Zhuhai Clinical Medical College of Jinan University, Zhuhai, China; ^2^Department of Breast Surgery Ward, Zhuhai People’s Hospital (The Affiliated Hospital of Beijing Institute of Technology, Zhuhai Clinical Medical College of Jinan University, Zhuhai, China; ^3^Zhuhai Clinical Medical College of Jinan University (Zhuhai People’s Hospital, The Affiliated Hospital of Beijing Institute of Technology), Guangzhou, China

**Keywords:** cross-sectional study, body roundness index, obesity, NHANES, thyroid hormones

## Abstract

**Background:**

The correlation between visceral adipose tissue and thyroid hormones is debated, and the conventional body mass index (BMI) is insufficient for differentiating fat distribution patterns. This study investigates the nonlinear relationship and threshold effects of the Body Roundness Index (BRI), a geometric metric of visceral fat (BRI = 364.2–365.5 × [1 – (waist circumference/2π)^2^/(0.5 × √height)^2^]), on thyroid hormone levels, hypothesizing that BRI influences thyroid hormone concentrations through a specific threshold.

**Methods:**

This study analyzes cross-sectional data from 10,086 participants in the National Health and Nutrition Examination Survey (NHANES) obtained between 2007 and 2012. Participants underwent anthropometric measurements and thyroid hormone assessments. We employed multiple linear and piecewise regressions to examine associations between BRI and the following thyroid hormones: free triiodothyronine (FT3), total triiodothyronine (TT3), free thyroxine (FT4), total thyroxine (TT4), and thyroid-stimulating hormone (TSH). We assessed nonlinearity and threshold effects and reported 95% confidence intervals and *p*-values.

**Results:**

The median age of participants was 43 years, with a BRI ranging from 0.77 to 19.33. After adjustments, a positive correlation was found between BRI and both TT3 (β = 0.95, 95% CI: 0.68–1.23) and TT4 (β = 0.06, 95% CI: 0.04–0.08). In contrast, a negative correlation was observed between BRI and FT4 (β = –0.03, 95% CI: –0.04 to –0.01). Threshold analysis revealed that when BRI was below 7.21, FT3 and TT3 increased with rising BRI, but this effect weakened or reversed beyond this threshold.

**Conclusion:**

In the American population, BRI is associated with non-linear relationships and threshold effects regarding thyroid hormone levels. Positive correlations exist between BRI and TT3/TT4, while a negative correlation is noted with FT4. Moreover, the dynamic threshold effect of BRI on FT3 and TSH indicates that visceral fat distribution characteristics should be considered when evaluating thyroid hormones.

## 1 Introduction

Thyroid hormones play a pivotal role in regulating energy metabolism within the body. This regulatory process is achieved through the hypothalamic–pituitary-thyroid (HPT) axis (1). FT3 and FT4 have been demonstrated to play a direct role in the regulation of basal metabolic rate, lipid oxidation, and the maintenance of cardiovascular function (2). Research indicates a significant association between hypothyroidism (low FT3/FT4) and an elevated risk of coronary heart disease (3). Conversely, elevated thyroid hormone levels (e.g., in hyperthyroidism) have been associated with the development of arrhythmias (4) and heart failure (5). Furthermore, TSH regulates the synthesis of thyroid hormones through a negative feedback mechanism. Abnormal fluctuations in TSH levels, such as those observed in obese populations, often reflect compensatory adaptations or pathological damage to the HPT axis (6).

Obesity, defined by a BMI of ≥30 kg/m^2^, is a pervasive global health concern that exhibits a multifaceted, bidirectional relationship with thyroid dysfunction. Conventional wisdom posits that hypothyroidism fosters adipogenesis by diminishing metabolic rate (7, 8). However, recent studies have found that individuals with obesity often present with elevated TSH levels and increased peripheral conversion of T3 (9), indicating that adipose tissue may interfere with the HPT axis through inflammatory factors or adipokines (10). Moreover, the interplay between obesity and its concomitant health complications is a subject that is garnering mounting attention from the research community. For instance, research has demonstrated a substantial correlation between BRI and overactive bladder syndrome (11), underscoring the significance of BRI in evaluating diverse health indicators in obese patients. Furthermore, research has indicated a substantial association between cardiovascular health and symptoms of obstructive sleep apnea (12), thereby suggesting that obesity may potentially influence disease progression through these metrics. It is important to acknowledge the significant limitations inherent in studies based on BMI: The BMI does not differentiate between the distribution of subcutaneous and visceral fat (13). Visceral fat accumulation is more likely to trigger insulin resistance and chronic inflammation (14, 15), potentially affecting thyroid function via mechanisms independent of BMI. This contradiction suggests that traditional anthropometric measurements are inadequate for elucidating the role of fat distribution heterogeneity in the phase-specific regulation of thyroid hormones.

The BRI is a metric used to quantify the proportion of visceral fat. It does so by integrating the dynamic relationship between waist circumference and height. The BRI can predict metabolic complications (16). In comparison with BMI, BRI has been demonstrated to exhibit superior sensitivity and specificity in the evaluation of diseases associated with abdominal obesity, including non-alcoholic fatty liver disease and cardiovascular risks (17, 18). While there is a mutual influence of BRI on other diseases and indicators, extant studies have largely focused on the linear association between BRI and metabolic syndrome and have not explored its non-linear dynamic characteristics concerning the thyroid hormone profile. Furthermore, the question of whether BRI reflects the metabolic threshold effect of visceral fat accumulation through geometric features, thereby affecting the compensation and decompensation transitions of thyroid function, remains under-researched.

The objective of this study is to examine the impact of BRI on thyroid hormone levels, with the central research question being: The central question guiding this study is whether a specific threshold exists for the impact of BRI on thyroid hormone levels. The inquiry posed pertains to the variability of the impact across diverse gender demographics and age ranges. The following research hypotheses are hereby proposed: Firstly, it is posited that a critical threshold (K) exists below which BRI is positively correlated with FT3 and TT3, while FT4 and TSH are negatively correlated. Secondly, it is further posited that beyond this threshold, the pattern of BRI’s influence on thyroid hormone levels will change significantly. To achieve this goal, this study will: The following three tasks must be completed in order to achieve the desired results: Identification of the nonlinear relationships between BRI and FT3, TT3, FT4, TT4, and TSH. Analysis of the impact of the BRI threshold (K) on thyroid hormones. Further investigation of the differences in correlations among different genders and age groups. The results of this study will enhance the understanding of the relationship between obesity and thyroid function, providing new insights for optimizing thyroid function screening processes in obese patients. Moreover, the findings of this study will provide significant theoretical underpinnings for future investigations into the mechanisms by which obesity impacts thyroid health. These findings could have substantial ramifications for the development of individual treatment plans and public health policy.

## 2 Methods

### 2.1 Study design

The data utilized in this study is derived from the publicly available NHANES dataset for the period from 2007 to 2012. The NHANES is a nationally representative health survey that collects information regarding demographics, health, and nutrition in order to assess the health and nutritional status of U.S. residents. This study utilizes NHANES data to examine the cross-sectional associations between BRI and thyroid hormone levels, with all data accessible through the official NHANES website.

### 2.2 Study population

**Figure 1:**
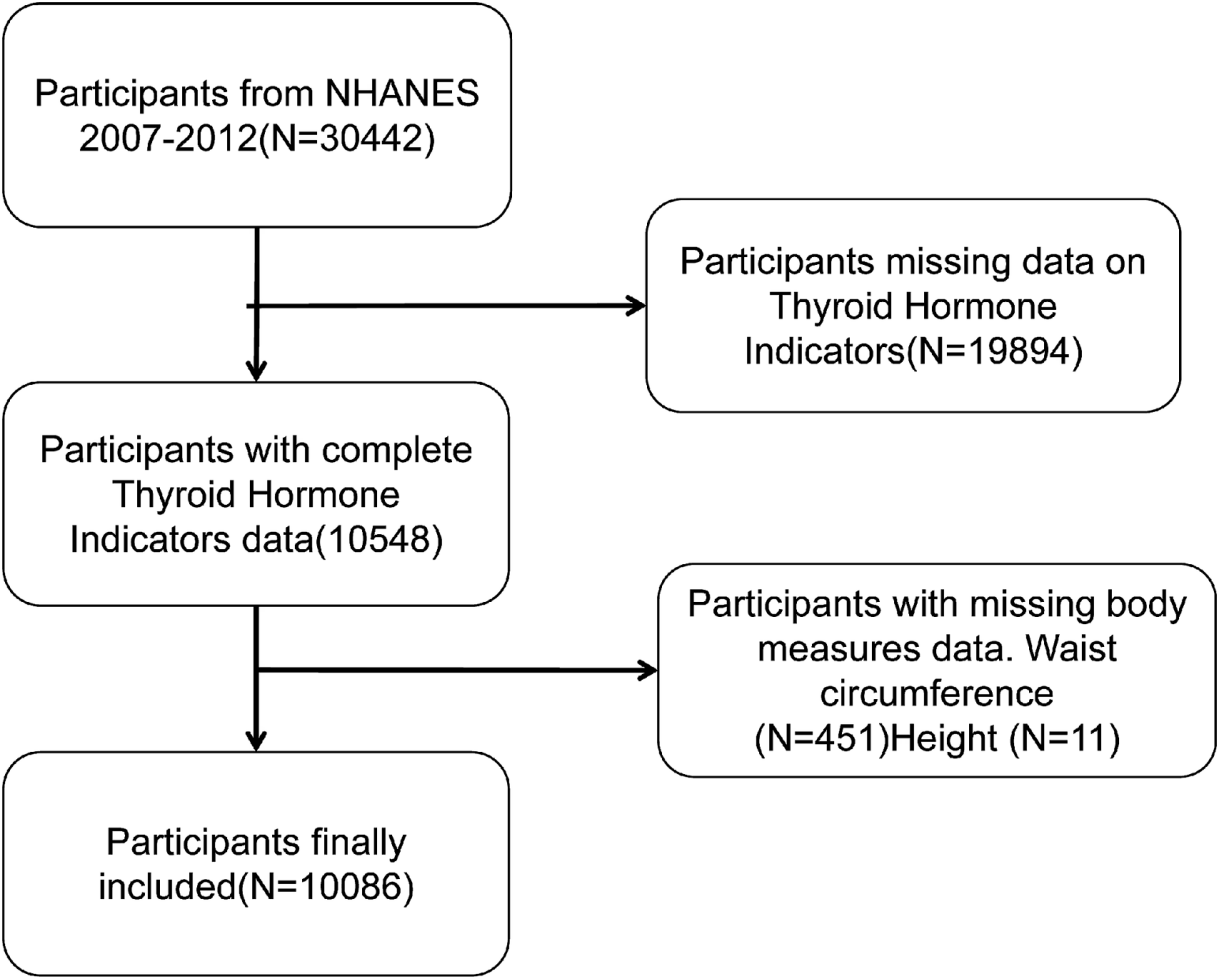
The flowchart of participant selection.

The present study’s participants were drawn from the NHANES database for the years 2007–2012, and they had complete waist circumference, height measurement data, and thyroid hormone test results. The following exclusion criteria were employed: Firstly, there is an absence of data regarding thyroid hormones (*n* = 19,894). Secondly, waist circumference and height data are not available for 462 subjects. Following the screening process, a total of 10,086 participants were deemed to have met the inclusion criteria (for a detailed exposition of the screening process, refer to Figure 1). The rationale behind the selection of this population was to safeguard the integrity and reliability of the data, thereby facilitating an accurate assessment of the relationship between BRI and thyroid hormone levels.

## 3 Variable definitions and measurements

### 3.1 Thyroid hormone indicators

Thyroid hormone levels were measured through NHANES laboratory tests, including FT3, TT3, FT4, TT4, and TSH. The following detection methods were employed: The measurement of FT3, TT3, and TT4 was conducted using a competitive binding immunoassay, while FT4 was measured via a two-step enzyme immunoassay. The assessment of TSH was performed using a third-generation two-site immunoassay, also known as a “sandwich” assay. For more detailed information regarding the laboratory methods, please refer to the official NHANES website.

### 3.2 BRI

BRI is calculated based on waist circumference (WC) and height, with all measurements performed by trained professionals at the Mobile Examination Center (MEC). The formula is as follows (16):


$$\mathrm{BRI}=364.2-365.5\times \sqrt{1-{\left(\mathrm{WC}(m)/2\times \pi \times h\text{eight}(m)/2\right)}^{2}}$$

### 3.3 Covariates

The set of covariates incorporated in the present study encompasses age, sex, race, educational attainment, the poverty-to-income ratio (PIR), hypertension status, diabetes status, specific dietary requirements, total food intake, along with thyroid peroxidase antibodies (TPOAB) and thyroglobulin (TG). The selection of these covariates was based on a review of the relevant literature, particularly studies related to BRI and thyroid hormones, ensuring coverage of key factors that may influence thyroid hormone levels (19–21).

Furthermore, to mitigate the potential for confounding bias, we excluded covariates with missing data exceeding 15% (22). With regard to the particulars of variable definitions, hypertension status was ascertained through the survey question, “Has a doctor told you that you have high blood pressure?” Participants who responded in the affirmative were designated as having hypertension. The identification of diabetes status was conducted using the same method. Furthermore, the evaluation of specialized dietary regimens was derived from the dietary behavior questionnaire inquiry, “Are you currently adhering to any specialized dietary regimen? (e.g., Mediterranean diet?)” Participants who responded in the affirmative were designated as adherents to a specialized dietary regimen. The total food intake (TI) of the subjects was calculated based on a 24-h dietary recall method, which included the total weight of all foods and beverages consumed. Furthermore, the laboratory testing module encompassed the collection of indicators, including total cholesterol and high-density lipoprotein cholesterol.

### 3.4 Statistical analysis

Given the failure of all thyroid hormone variables (FT3, FT4, TT3, TT4, TSH) to satisfy the normality test (Shapiro–Wilk *p* < 0.001), non-parametric tests were employed. For the purpose of conducting intergroup comparisons, the Kruskal-Wallis test was employed, with pairwise comparisons subsequently executed using Dunn’s method in instances where significant disparities were identified. The Mann–Whitney U test was employed to conduct comparisons between two groups. Linear regression models were established to explore the association between BRI and thyroid hormones, with covariates progressively adjusted. Model 1 was unadjusted; Model 2 was adjusted for age, sex, and race; and Model 3 was further augmented with socioeconomic factors (education level, poverty-to-income ratio), lifestyle factors (special diet and total food intake), metabolic indicators (hypertension, diabetes, total cholesterol, high-density lipoprotein cholesterol), as well as TPOAB and TG. In order to test for a non-linear relationship between BRI and thyroid hormones, penalized spline smoothing curves were fitted, and the significance of the non-linear trends was assessed through likelihood ratio tests. The threshold effect analysis was conducted using piecewise regression models to identify the inflection point (K) in the association between BRI and thyroid hormones. The significance of the threshold effect was evaluated by the log-likelihood ratio test. Subgroup analyses were conducted to assess the heterogeneity of the association between BRI and thyroid hormones. The significance of differences between subgroups was evaluated using interaction *p*-values. All statistical analyses were conducted using EmpowerStats software (versions 4.2 and 2.0), with *p* < 0.05 considered statistically significant.

## 4 Results

As illustrated in Table 1, the present study encompassed a total of 10,086 participants, with 50.05% identifying as male and 49.95% as female. The median age of the participants was determined to be 43 years, with a range extending from 12 to 80 years. Notably, the majority of the participants were non-Hispanic White, accounting for 43.23% of the total sample. The analysis revealed that the sample sizes across the cohorts were similar when grouped by BRI quartiles (Q1: 2,522 participants, 0.77–3.38; Q2: 2,521 participants, 3.38–4.79; Q3: 2,521 participants, 4.79–6.34; Q4: 2,522 participants, 6.34–19.33). The median levels of FT3 (3.16–3.30 pg./mL), FT4 (0.80 ng/dL), TSH (1.38–1.74 mIU/L), TT3 (112–118 ng/dL), and TT4 (7.46–8.10 μg/dL) exhibited significant differences among the quartile groups. The prevalence of hypertension (52.33%), diabetes (23.24%), and special diet patterns (21.25%) was highest in the Q4 group, while a higher poverty-to-income ratio (median 2.26) and a greater proportion of college education (27.79%) were primarily found in the low BRI group (Q1-Q2). The proportion of non-Hispanic Black participants was highest in Q1 (24.46%), while the proportion of Mexican American participants was greatest in Q4 (19.79%).

**Table 1:** Characteristics of participants by BRI quartiles.

Characteristics	Overall	BRI quartile				*P*-value
		Q1 (0.77–3.38)	Q2 (3.38–4.79)	Q3 (4.79–6.34)	Q4 (6.34–19.33)	
*N*	10,086	2,522	2,521	2,521	2,522	
FT3 (pg/ml)	3.20 (1.73–28.84)	3.30 (1.73–17.50)	3.20 (1.80–28.84)	3.12 (1.86–6.84)	3.16 (1.80–14.00)	<0.001
FT4 (ng/dl)	0.80 (0.10–4.80)	0.80 (0.10–3.70)	0.80 (0.20–4.80)	0.80 (0.30–2.60)	0.80 (0.18–4.70)	0.005
TSH (miu/l)	1.53 (0.00–280.76)	1.38 (0.00–280.76)	1.48 (0.00–50.47)	1.55 (0.00–97.01)	1.74 (0.00–69.84)	0.015
TT3 (ng/dl)	114.00 (37.00–707.00)	118.00 (54.00–562.00)	113.00 (38.00–632.00)	112.00 (37.00–311.00)	113.00 (50.00–707.00)	<0.001
TT4 (ug/dl)	7.70 (0.40–27.60)	7.46 (1.50–23.10)	7.60 (2.00–27.60)	7.80 (2.00–18.90)	8.10 (0.40–21.50)	<0.001
TG (ng/ml)	9.75 (0.07–4461.00)	9.50 (0.07–368.73)	9.24 (0.07–1259.61)	9.68 (0.07–2038.94)	10.89 (0.07–4461.00)	<0.001
TPOAB (iu/ml)	0.60 (0.10–2376.00)	0.60 (0.10–942.00)	0.60 (0.10–991.40)	0.60 (0.10–910.40)	0.60 (0.10–2376.00)	<0.001
Age (years)	43.00 (12.00–80.00)	22.00 (12.00–80.00)	43.00 (12.00–80.00)	51.00 (12.00–80.00)	54.00 (12.00–80.00)	<0.001
PIR	1.99 (0.00–5.00)	1.96 (0.00–5.00)	2.26 (0.00–5.00)	2.06 (0.00–5.00)	1.71 (0.00–5.00)	<0.001
GENDER (%)						<0.001
Male	5,048 (50.05%)	1,362 (54.00%)	1,339 (53.11%)	1,330 (52.76%)	1,017 (40.33%)	
Female	5,038 (49.95%)	1,160 (46.00%)	1,182 (46.89%)	1,191 (47.24%)	1,505 (59.67%)	
Race/Hispanic origin (%)						<0.001
Mexican American people	1782 (17.67%)	338 (13.40%)	433 (17.18%)	512 (20.31%)	499 (19.79%)	
Other Hispanic people	1,145 (11.35%)	238 (9.44%)	287 (11.38%)	329 (13.05%)	291 (11.54%)	
Non-Hispanic White people	4,360 (43.23%)	1,055 (41.83%)	1,136 (45.06%)	1,081 (42.88%)	1,088 (43.14%)	
Non-Hispanic Black people	2078 (20.60%)	617 (24.46%)	458 (18.17%)	438 (17.37%)	565 (22.40%)	
Other Race—Including Multi-Racial people	721 (7.15%)	274 (10.86%)	207 (8.21%)	161 (6.39%)	79 (3.13%)	
Education level, *n*, (%)						<0.001
Less than 9th grade	1,017 (12.21%)	71 (4.96%)	220 (10.10%)	350 (14.93%)	376 (15.84%)	
9-11th grade (Includes 12th grade with no diploma)	1,379 (16.56%)	224 (15.64%)	303 (13.91%)	390 (16.63%)	462 (19.47%)	
High school graduate/GED or equivalent	1941 (23.30%)	333 (23.25%)	486 (22.30%)	563 (24.01%)	559 (23.56%)	
Some college or AA degree	2,284 (27.42%)	406 (28.35%)	599 (27.49%)	619 (26.40%)	660 (27.81%)	
College graduate or above	1708 (20.51%)	398 (27.79%)	571 (26.20%)	423 (18.04%)	316 (13.32%)	
Hypertension (%)						<0.001
Yes	2,940 (31.97%)	192 (9.74%)	575 (24.43%)	892 (36.83%)	1,281 (52.33%)	
No	6,256 (68.03%)	1780 (90.26%)	1779 (75.57%)	1,530 (63.17%)	1,167 (47.67%)	
Diabetes (%)						<0.001
Yes	1,015 (10.23%)	32 (1.28%)	144 (5.78%)	271 (10.94%)	568 (23.24%)	
No	8,906 (89.77%)	2,475 (98.72%)	2,349 (94.22%)	2,206 (89.06%)	1876 (76.76%)	
Other special diet (%)						<0.001
Yes	1,264 (12.97%)	131 (5.38%)	265 (10.89%)	350 (14.36%)	518 (21.25%)	
No	8,478 (87.03%)	2,302 (94.62%)	2,168 (89.11%)	2088 (85.64%)	1920 (78.75%)	
Total food intake (g)	22004.56 (82.92–263324.74)	20384.30 (296.10–263324.74)	23168.64 (522.08–169261.28)	22336.10 (223.05–220604.74)	22038.53 (82.92–137762.55)	0.003
Total cholesterol (mmol/L)	4.78 (1.94–11.90)	4.32 (2.30–11.17)	4.89 (2.07–11.84)	5.07 (2.17–9.98)	4.97 (1.94–11.90)	<0.001
Direct HDL-cholesterol (mmol/L)	1.29 (0.18–4.63)	1.45 (0.54–3.88)	1.32 (0.41–4.63)	1.22 (0.18–3.34)	1.16 (0.28–2.84)	<0.001

^a^The results of the normality tests indicated that all thyroid hormone variables (FT3, FT4, TT3, TT4, TSH, TG, TPOAB) exhibited significant deviation from a normal distribution (Shapiro–Wilk P < 0.001). Consequently, non-parametric tests were employed. Comparisons between multiple groups were conducted using the Kruskal-Wallis test, with pairwise comparisons performed using Dunn’s method when significant differences were identified. Comparisons between two groups utilized the Mann–Whitney U test. ^b^Number and proportion of missing data: PIR (*n* = 854, 8.47%), special diet (*n* = 344, 3.41%), total food intake (*n* = 352, 3.49%), total cholesterol (*n* = 1, 0.01%), direct HDL-C (*n* = 1, 0.01%), TG (*n* = 4, 0.04%), TPOAB (*n* = 81, 0.80%), hypertension (*n* = 890, 8.82%), diabetes (*n* = 165, 1.64%). ^c^BRI (Body Roundness Index), Q (Quartile), PIR (Poverty-Income Ratio), FT3 (Free Triiodothyronine), TT3 (Total Triiodothyronine), FT4 (Free Thyroxine), TT4 (Total Thyroxine), TSH (Thyroid-Stimulating Hormone), TPOAB (Thyroid Peroxidase Antibodies), TG (Thyroglobulin), HDL-C (High-Density Lipoprotein Cholesterol). ^d^Statistical significance markers: *p < 0.05, **p < 0.01, ***p < 0.001.

### 4.1 Associations between BRI and thyroid hormones

As illustrated in Table 2, an examination of the relationship between BRI and thyroid hormones is presented across a range of adjusted models. In the fully adjusted model (Model 3), BRI demonstrated a positive linear association with TT3 (*β* = 0.95, 95% CI: 0.68–1.23, *p* < 0.0001) and TT4 (*β* = 0.06, 95% CI: 0.04–0.08), while exhibiting a negative linear association with FT4 (*β* = −0.03, 95% CI: −0.04 – −0.01). FT3 demonstrated a modest positive tendency in relation to BRI (*β* = 0.01, 95% CI: 0.00–0.01), while TSH exhibited an absence of statistically significant correlation (β = 0.03, 95% CI: −0.00–0.06). Quartile analysis revealed that as BRI quartiles increased, TT3 (Q4: *β* = 7). The 95% confidence interval (CI) for the proportionate change in the mean (*β*) was calculated to be from 5.76 to 9.54, and the trend *p*-value was determined to be less than 0.0001. Similarly, the mean of the thyroid stimulating hormone (TSH) (Q4) increased (β = 0.26, 95% CI: 0.14–0.39, trend *p*-value < 0.0001), while the mean of the free triiodothyronine (FT3) (Q4) decreased (*β* = −0.19, 95% CI: −0.33 – −0.06, trend *p*-value = 0.0015). The FT3 exhibited an upward trend, followed by a downward trend, with the highest quartile demonstrating a positive association in comparison to the reference group (Q4: β = 0.07, 95% CI: 0.03–0.11). However, a linear trend was not observed (trend *p*-value = 0.0006). The quartile analysis for TSH revealed no statistically significant dose–response relationship (trend *p*-value = 0.9395).

**Table 2:** The associations between BRI and thyroid hormones.

Characteristics	Model 1 [β (95% CI)]	Model 2 [β (95% CI)]	Model 3 [β (95% CI)]
FT3 (pg/ml)			
BRI (continuous)	−0.03 (−0.03, −0.02)	0.01 (0.01, 0.02)	0.01 (0.00, 0.01)
BRI (quartile)			
Quartile1	Ref.	Ref.	Ref.
Quartile2	−0.14 (−0.17, −0.11)	0.03 (−0.01, 0.06)	0.03 (−0.00, 0.07)
Quartile3	−0.20 (−0.23, −0.17)	0.03 (0.00, 0.06)	0.03 (−0.01, 0.07)
Quartile4	−0.19 (−0.22, −0.16)	0.08 (0.05, 0.12)	0.07 (0.03, 0.11)
P for trend	−0.06 (−0.07, −0.05)	0.03 (0.02, 0.04)	0.02 (0.01, 0.03)
TT3 (ng/dl)			
BRI (continuous)	−0.65 (−0.88, −0.42)	0.95 (0.72, 1.18)	0.95 (0.68, 1.23)
BRI (quartile)			
Quartile1	Ref.	Ref.	Ref.
Quartile2	−4.92 (−6.39, −3.45)	2.41 (0.98, 3.84)	3.22 (1.52, 4.93)
Quartile3	−6.20 (−7.67, −4.73)	4.28 (2.78, 5.78)	4.82 (3.02, 6.61)
Quartile4	−4.59 (−6.05, −3.12)	7.12 (5.60, 8.64)	7.65 (5.76, 9.54)
P for trend	−1.50 (−1.97, −1.04)	2.33 (1.85, 2.81)	2.42 (1.83, 3.01)
FT4 (ng/dl)			
BRI (continuous)	−0.01 (−0.02, 0.00)	−0.03 (−0.04, −0.01)	−0.03 (−0.04, −0.01)
BRI (quartile)			
Quartile1	Ref.	Ref.	Ref.
Quartile2	−0.05 (−0.14, 0.05)	−0.16 (−0.25, −0.06)	−0.09 (−0.21, 0.02)
Quartile3	−0.17 (−0.26, −0.07)	−0.32 (−0.42, −0.22)	−0.23 (−0.36, −0.11)
Quartile4	−0.06 (−0.15, 0.04)	−0.21 (−0.32, −0.11)	−0.19 (−0.33, −0.06)
P for trend	−0.03 (−0.06, 0.00)	−0.08 (−0.11, −0.04)	−0.07 (−0.11, −0.03),
TT4 (ug/dl)			
BRI (continuous)	0.11 (0.10, 0.13)	0.09 (0.07, 0.10)	0.06 (0.04, 0.08)
BRI (quartile)			
Quartile1	Ref.	Ref.	Ref.
Quartile2	0.16 (0.07, 0.26)	0.08 (−0.01, 0.18)	0.04 (−0.07, 0.16)
Quartile3	0.35 (0.26, 0.44)	0.23 (0.13, 0.32)	0.14 (0.01, 0.26)
Quartile4	0.62 (0.53, 0.71)	0.44 (0.34, 0.54)	0.26 (0.14, 0.39)
P for trend	0.20 (0.18, 0.23)	0.15 (0.12, 0.18)	0.09 (0.05, 0.13)
TSH (miu/u)			
BRI (continuous)	0.07 (0.04, 0.10)	0.04 (0.00, 0.07)	0.03 (−0.00, 0.06)
BRI (quartile)			
Quartile1	Ref.	Ref.	Ref.
Quartile2	0.07 (−0.15, 0.29)	−0.11 (−0.34, 0.12)	−0.12 (−0.33, 0.08)
Quartile3	0.18 (−0.04, 0.40)	−0.07 (−0.31, 0.17)	−0.15 (−0.37, 0.07)
Quartile4	0.34 (0.12, 0.56)	0.06 (−0.18, 0.30)	−0.04 (−0.26, 0.19)
P for trend	0.11 (0.04, 0.18)	0.03 (−0.05, 0.10)	−0.00 (−0.07, 0.07)

^a^Model definitions: Model 1: Unadjusted covariates (crude association analysis); Model 2: Adjusted for age, sex, and race; Model 3: Further adjusted for education level, hypertension, poverty-to-income ratio (PIR), diabetes, triglycerides (TG), thyroid peroxidase antibodies (TPOAB), special diet, total food intake (g), total cholesterol (mmol/L), and direct HDL-C. ^b^Trend tests (P for trend): Based on the analysis of the ordered variable of BRI quartiles. ^c^BRI: Body Roundness Index; PIR: Poverty-Income Ratio; FT3: Free Triiodothyronine; TT3: Total Triiodothyronine; FT4: Free Thyroxine; TT4: Total Thyroxine; TSH: Thyroid-Stimulating Hormone. ^d^Statistical significance markers: *p < 0.05, **p < 0.01, ***p < 0.001.

### 4.2 Smooth curve fitting and threshold effect analysis

**Figure 2:**
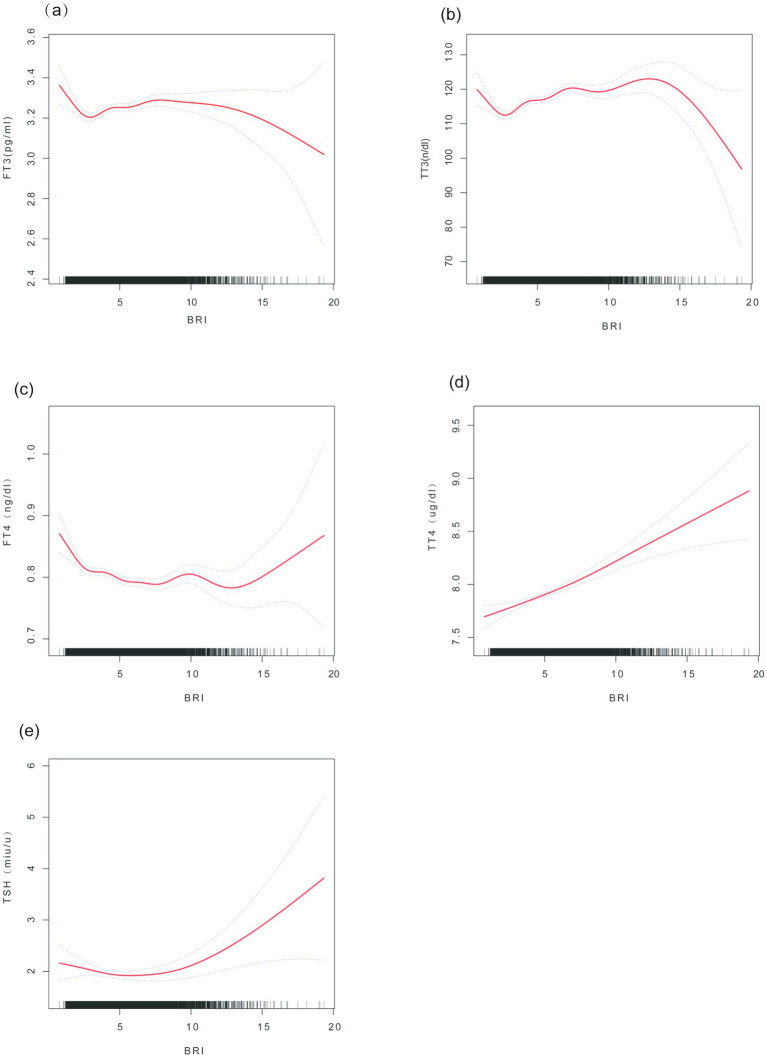
Smooth curve fitting of the association between BRI and thyroid hormones. **(a)** FT3 vs. BRI; **(b)** FT4 vs. BRI; **(c)** TSH vs. BRI; **(d)** TT3 vs. BRI; **(e)** TT4 vs. BRI. Note: The curves are based on Model 3 (adjusted for age, sex, race, education level, hypertension, poverty-to-income ratio (PIR), diabetes, triglycerides (TG), thyroid peroxidase antibodies (TPOAB), diet, total cholesterol, HDL-C, etc.). Axis units: BRI; thyroid hormones are measured in pg./ml (FT3), ng/dl (FT4), mIU/L (TSH), ng/dl (TT3), and μg/dl (TT4).

The penalized spline smoothing curve (red solid line) in Figure 2 illustrates the non-linear associations between BRI and thyroid function indicators (FT3, TT3, FT4, TT4, TSH) under the fully adjusted model (Model 3), with the shaded area representing the 95% confidence interval. The likelihood ratio tests indicated significant non-linear trends for FT3 (*p* = 0.007), TT3 (*p* < 0.001), FT4 (*p* = 0.002), and TSH (*p* < 0.001), while no significant non-linearity was observed for TT4 (*p* = 0.142). The associations of FT3 and TT3 with BRI demonstrate a trend of initially increasing, followed by leveling off or reversing; FT4 decreases with increasing BRI before stabilizing, whereas TSH first decreases and then increases.

Threshold effect analysis (Table 3) revealed the inflection point (K) in the association between BRI and thyroid hormones. In the fully adjusted model, when BRI is below the K value, each unit increase in BRI significantly raises FT3 [*β* = 0.18 (0.09, 0.27)] and TT3 [β = 1.78 (1.33, 2.22)], while FT4 [β = −0.09 (−0.13, −0.04)] declines. Conversely, when BRI is above the K value, the effects of increasing BRI on FT3 [β = −0.07 (−0.19, 0.06)] and TT3 [β = −0.22 (−0.79, 0.34)] are weakened or reversed, but TT4 [β = 0.06 (0.04, 0.08)] and TSH [β = 0.32 (0.19, 0.45)] significantly increase. The log-likelihood ratio test provides substantial evidence in support of the threshold effects for FT3, TT3, FT4, and TSH (all *p* < 0.05), while TT4 demonstrates no significant threshold effect (*p* = 0.142).

**Table 3:** Threshold effect analysis between BRI and thyroid hormones.

Characteristics	FT3 (pg/ml)	TT3 (ng/dl)	FT4 (ng/dl)	TT4 (ug/dl)	TSH (miu/u)
Breakpoint (K)	7.21	7.06	5.61	2.7	9.54
Effect in the segment < K	0.18 (0.09, 0.27)	1.78 (1.33, 2.22)	−0.09 (−0.13, −0.04)	−0.13 (−0.39, 0.12)	−0.02 (−0.06, 0.02)
Effect in the segment > K	−0.07 (−0.19, 0.06)	−0.22 (−0.79, 0.34)	0.01 (−0.02, 0.04)	0.06 (0.04, 0.08)	0.32 (0.19, 0.45)
Log-likelihood ratio test	0.007	<0.001	0.002	0.142	<0.001

^a^All models adjusted for age, sex, race, education level, hypertension, poverty-to-income ratio (PIR), diabetes, thyroglobulin (TG, ng/ml), thyroid peroxidase antibodies (TPOAB, IU/ml), special diet, total food intake (g), total cholesterol (total Cholesterol, mmol/L), and direct high-density lipoprotein cholesterol (direct HDL-C, mmol/L). ^b^Units Explanation: FT3: Free Triiodothyronine (pg/ml); TT3: Total Triiodothyronine (ng/dl); FT4: Free Thyroxine (ng/dl); TT4: Total Thyroxine (μg/dl); TSH: Thyroid-Stimulating Hormone (mIU/L). ^c^BRI: Body Roundness Index. *p < 0.05, **p < 0.01, ***p < 0.001.

### 4.3 Subgroup analysis

As demonstrated in Table 4, BRI exhibits a consistent positive association with FT3, TT3, and TT4 in the majority of subgroups. Specifically, a 1-unit increase in BRI is associated with a 0.09 pg./mL rise in FT3 (95% CI: 0.03–0.15), a 1.12 ng/dL rise in TT3 (95% CI: 0.78–1.46), and a 0.07 μg/dL rise in TT4 (95% CI: 0.04–0.10). A more pronounced correlation was observed in female subjects (FT3 interaction *p* = 0.0035), among those with elevated total food intake (TT3 interaction *p* = 0.0033), and in individuals with a college education (TT3 interaction *p* = 0.0175). Conversely, BRI exhibited a negative correlation with FT4 in males (*β* = −0.04, 95% CI: −0.07 to −0.01) and among Hispanic Americans (β = −0.05, 95% CI: −0.08 to −0.01). However, these interactions did not attain statistical significance (*p* > 0.05). No significant heterogeneity was observed for TSH across all subgroups.

**Table 4:** Subgroup analysis of the association between BRI and thyroid hormones.

Subgroup	FT3 [β (95%CI)]	FT4 [β (95%CI)]	TSH [β (95%CI)]	TT3 [β (95%CI)]	TT4 [β (95%CI)]
Sex: male	0.02 (−0.04, 0.09)	−0.04 (−0.07, −0.01)	0.03 (−0.01, 0.07)	1.02 (0.58, 1.47)	0.04 (0.02, 0.07)
Sex: female	0.12 (0.06, 0.19)	−0.02 (−0.06, 0.01)	0.05 (−0.01, 0.12)	1.06 (0.73, 1.39)	0.08 (0.05, 0.12)
P for interaction	0.0035	0.5681	0.5236	0.8800	0.0202
Age:≤39 years	0.05 (−0.03, 0.12)	−0.04 (−0.07, −0.01)	0.08 (0.01, 0.16)	1.36 (0.89, 1.83)	0.07 (0.03, 0.11)
Age:40-60 years	0.11 (0.04, 0.18)	−0.02 (−0.04, 0.01)	0.02 (−0.04, 0.09)	1.02 (0.60, 1.43)	0.07 (0.04, 0.11)
Age:>60 years	0.12 (−0.02, 0.25)	−0.03 (−0.08, 0.01)	0.00 (−0.06, 0.06)	0.51 (−0.16, 1.18)	0.05 (0.01, 0.10)
P for interaction	0.3841	0.1658	0.2001	0.1057	0.7728
PIR:≤1.3	0.03 (−0.05, 0.12)	−0.05 (−0.09, −0.01)	0.07 (0.03, 0.11)	0.90 (0.40, 1.39)	0.06 (0.03, 0.10)
PIR:1.3–3.5	0.06 (−0.02, 0.14)	−0.02 (−0.05, 0.00)	0.02 (−0.07, 0.10)	0.95 (0.39, 1.50)	0.08 (0.04, 0.11)
PIR:>3.5	0.15 (0.06, 0.24)	−0.02 (−0.05, 0.01)	0.06 (−0.01, 0.12)	1.27 (0.70, 1.84)	0.06 (0.02, 0.10)
P for interaction	0.0864	0.3345	0.5525	0.7101	0.7512
Mexican American	−0.03 (−0.17, 0.11)	−0.05 (−0.08, −0.01)	0.05 (−0.02, 0.12)	1.25 (0.63, 1.87)	0.05 (−0.00, 0.09)
Other Hispanic	0.13 (−0.01, 0.27)	−0.06 (−0.12, −0.01)	0.26 (−0.07, 0.60)	0.82 (−0.19, 1.83)	0.02 (−0.06, 0.10)
Non-Hispanic White	0.09 (0.02, 0.16)	−0.03 (−0.06, −0.01)	0.03 (−0.02, 0.09)	0.96 (0.63, 1.29)	0.07 (0.03, 0.10)
Non-Hispanic Black	0.06 (−0.06, 0.17)	−0.00 (−0.04, 0.04)	0.03 (−0.04, 0.10)	0.94 (0.24, 1.64)	0.09 (0.05, 0.13)
Other race	0.21 (−0.12, 0.53)	−0.03 (−0.13, 0.07)	0.01 (−0.06, 0.07)	2.65 (0.61, 4.70)	0.09 (0.00, 0.19)
P for interaction	0.3171	0.2146	0.6314	0.5050	0.2670
Less than 9th grade	0.03 (−0.07, 0.14)	0.00 (−0.06, 0.06)	0.04 (−0.01, 0.09)	0.14 (−0.73, 1.02)	0.09 (0.02, 0.15)
9-11th grade	0.04 (−0.04, 0.12)	−0.00 (−0.03, 0.02)	0.04 (−0.02, 0.10)	1.10 (0.52, 1.68)	0.09 (0.05, 0.13)
High school graduate	−0.04 (−0.13, 0.06)	−0.07 (−0.11, −0.02)	0.03 (−0.06, 0.12)	0.63 (0.09, 1.16)	0.06 (0.03, 0.10)
Some college	0.13 (0.04, 0.22)	−0.03 (−0.07, 0.01)	0.01 (−0.08, 0.11)	0.96 (0.41, 1.50)	0.05 (0.01, 0.09)
College graduate	0.21 (0.10, 0.32)	−0.02 (−0.05, 0.02)	0.11 (0.00, 0.21)	1.94 (1.22, 2.66)	0.08 (0.01, 0.14)
P for interaction	0.0003	0.0781	0.7414	0.0175	0.6638
Other special diet	0.01 (−0.10, 0.11)	−0.02 (−0.08, 0.04)	0.08 (−0.01, 0.17)	0.43 (−0.30, 1.16)	0.05 (−0.01, 0.10)
Non—other special diet	0.10 (0.05, 0.16)	−0.03 (−0.06, −0.01)	0.04 (−0.02, 0.09)	1.17 (0.88, 1.47)	0.07 (0.04, 0.10)
P for interaction	0.0562	0.7816	0.3833	0.0520	0.4278
Low total food intake	0.03 (−0.04, 0.11)	−0.05 (−0.08, −0.02)	0.07 (0.00, 0.14)	0.57 (0.17, 0.97)	0.04 (0.00, 0.07)
High total food intake	0.12 (0.05, 0.20)	−0.02 (−0.05, 0.01)	0.02 (−0.03, 0.08)	1.37 (0.98, 1.77)	0.09 (0.06, 0.12)
P for interaction	0.0711	0.1147	0.2062	0.0033	0.0078
Low HDL-cholesterol	0.09 (0.01, 0.18)	−0.01 (−0.03, 0.01)	0.04 (−0.00, 0.08)	1.19 (0.77, 1.61)	0.08 (0.05, 0.10)
High HDL-cholesterol	0.08 (0.01, 0.15)	−0.04 (−0.07, −0.02)	0.05 (−0.02, 0.12)	0.93 (0.59, 1.28)	0.06 (0.03, 0.09)
P for interaction	0.7868	0.0509	0.7936	0.2923	0.2111
Low TG	0.05 (−0.02, 0.12)	−0.03 (−0.07, 0.00)	0.08 (0.00, 0.16)	1.05 (0.74, 1.36)	0.06 (0.02, 0.09)
High TG	0.11 (0.05, 0.18)	−0.03 (−0.05, −0.00)	0.02 (−0.03, 0.07)	1.04 (0.60, 1.49)	0.07 (0.04, 0.11)
P for interaction	0.1130	0.7370	0.1399	0.9809	0.4929
Low TPOAB	0.08 (0.00, 0.15)	−0.03 (−0.06, 0.01)	0.03 (−0.00, 0.06)	0.96 (0.55, 1.38)	0.06 (0.03, 0.09)
High TPOAB	0.09 (0.03, 0.16)	−0.03 (−0.06, −0.00)	0.06 (−0.01, 0.13)	1.11 (0.62, 1.61)	0.07 (0.04, 0.10)
P for interaction	0.6366	0.8023	0.3265	0.6740	0.7815
HTN	0.07 (−0.01, 0.14)	−0.01 (−0.05, 0.02)	0.03 (−0.02, 0.09)	0.72 (0.22, 1.23)	0.08 (0.03, 0.12)
−HTN	0.10 (0.03, 0.16)	−0.04 (−0.07, −0.01)	0.05 (−0.01, 0.11)	1.22 (0.86, 1.57)	0.06 (0.03, 0.09)
P for interaction	0.4552	0.2346	0.6684	0.1131	0.6003
Diabetes	0.09 (−0.03, 0.20)	−0.01 (−0.10, 0.07)	0.02 (−0.28, 0.31)	0.20 (−0.62, 1.02)	0.10 (0.02, 0.18)
Non—diabetes	0.09 (0.02, 0.15)	−0.03 (−0.06, −0.01)	0.05 (0.00, 0.09)	1.16 (0.83, 1.49)	0.06 (0.04, 0.09)
P for interaction	0.9818	0.7111	0.8564	0.0353	0.4129
low total cholesterol	0.03 (−0.03, 0.10)	−0.03 (−0.05, 0.00)	0.07 (0.01, 0.12)	0.55 (0.18, 0.93)	0.05 (0.02, 0.09)
High total cholesterol	0.15 (0.07, 0.23)	−0.04 (−0.07, −0.00)	0.02 (−0.05, 0.08)	1.67 (1.22, 2.13)	0.09 (0.05, 0.12)
P for interaction	0.0110	0.6239	0.1904	0.0001	0.2173

^a^All models are based on the fully adjusted model (adjusted for age, sex, race, education level, poverty-to-income ratio (PIR), diabetes, total food intake [g], total cholesterol [mmol/L], high-density lipoprotein cholesterol [HDL-C, mmol/L], thyroid peroxidase antibodies [TPOAB, IU/ml], and hypertension). ^b^Data presentation: the results are expressed as regression coefficients (β) and their 95% confidence intervals (95% CI). Heterogeneity among subgroups is assessed using interaction *p* values, with a significance threshold of *p* < 0.05. ^c^BRI: Body Roundness Index; TSH: Thyroid-Stimulating Hormone; FT3/4: Free Triiodothyronine/Thyroxine; TT3/4: Total Triiodothyronine/Thyroxine; Q: Quartile; HTN: Hypertension;-HTN: Non-hypertensive.

Sensitivity analysis and preliminary analyses demonstrated a high degree of consistency in trends. Following the resolution of missing values for covariates through the implementation of dummy variables (23), the results of the supplementary analysis demonstrated a strong alignment with the primary findings, as illustrated in Supplementary Table S1. Specifically, the dummy variable method employed in the supplementary analysis effectively mitigated the biases introduced by missing data, ensuring the reliability of the results and enhancing the understanding of the relationship between BRI and thyroid hormones.

## 5 Discussion

This study, which is based on a large sample of data from the NHANES in the United States, reveals a non-linear association and threshold effect between the body roundness index (BRI) and thyroid hormone levels. The findings of the present study demonstrate a positive correlation between BRI and TT3 and TT4, a negative correlation between BRI and FT4, and the presence of dynamic thresholds in the effects of BRI on FT3 and TSH, with heterogeneity observed across different subgroups. It is noteworthy that when BRI is less than 7.21, FT3 increases with rising visceral fat. However, this increase ceases or even reverses when BRI exceeds 7.21. Furthermore, TSH levels significantly increase when BRI is greater than 9.54. These findings offer novel insights into the relationship between the geometric distribution characteristics of visceral fat and thyroid dysfunction.

In the course of the present discussion of literature comparison and research background, it was found that the majority of related studies focus on the relationship between obesity and its derived body parameters, as well as changes in thyroid hormones. Peter N. Taylor and colleagues conducted a cross-sectional study involving 3,014 children and adolescents, showing that higher BMI levels were associated with increased FT3 levels, while FT4 did not demonstrate significant changes. This phenomenon does not align entirely with the hormonal characteristics of conventional thyroid diseases, suggesting potential discrepancies in the findings. Concurrently, Xiaoyong Wen and his team introduced the weight-adjusted waist index (WWI) to assess the risk of central obesity and revealed its significant association with Hashimoto’s thyroiditis, particularly prominent in females (24). While this study introduces a novel measurement tool for central obesity, it underscores the association analysis between WWI and Hashimoto’s thyroiditis. In contrast, the present research focuses on the characteristics and variation patterns of thyroid hormones. Ranran Xu and colleagues conducted a cohort study in Wuhan, finding that individuals who are overweight or obese had higher levels of FT3 and FT3/FT4 ratios, while FT4 levels decreased. Conversely, individuals with a body weight below the standard had above-average FT4 levels and lower FT3 levels (25). The present study primarily focused on the relationship between weight status and thyroid hormones, which differs from the direction of our research. Additionally, Meng-Ting Tsou and colleagues provided clinical data from Taiwan indicating that individuals with subclinical hypothyroidism had significantly higher BRI compared to the healthy group, with this trend being particularly pronounced in female subjects (26). However, the present study did not further explore the direct relationship between BRI and thyroid hormone parameters. Sevin Demirdogdu et al. investigated 675 participants with BMI ≥ 30 and TSH levels between 0.4 and 4.5 mIU/L, and found no statistically significant correlation between TSH, FT3, FT4, and BRI (27), which contrasts with the findings of the present study. In addition, the team led by Gülsüm Gönülalan found that patients with hypothyroidism generally exhibited increased metabolic risk. The assessment of the body roundness index has the potential to facilitate the early identification of atypical fat distribution and central obesity, which may contribute to the prevention and management of cardiovascular complications (28). However, it is important to note that the study population in their research differs from that of our inquiry, which raises questions about the generalizability of their conclusions.

The present study explores the potential of BRI as a sensitive indicator of visceral fat, with the results supporting its effectiveness as a tool for measuring visceral fat accumulation. As demonstrated in prior *in vitro* and epidemiological studies, visceral fat has been shown to directly impact hypothalamic–pituitary-thyroid (HPT) axis signaling through the secretion of inflammatory factors, such as tumor necrosis factor-alpha (TNF-*α*) and interleukin-6 (IL-6). This process involves mechanisms such as leptin resistance, thereby interfering with thyroid hormone metabolism (29, 30). Despite the prevalence of waist circumference and waist-to-hip ratios in current research, these metrics often fall short in fully capturing the three-dimensional characteristics of visceral fat. The introduction of BRI, which incorporates waist circumference and height into a geometric formula, offers a more scientific representation of fat distribution within the body (16). Our study found a significant positive correlation between TT3 and BRI, with this association remaining statistically significant even after thorough adjustment for confounding factors (*β* = 0.95, *p* < 0.0001). This finding aligns with the observations reported by Zhao et al., who identified an independent correlation between TT3 levels and visceral fat area. Their findings suggest that for every 1 nmol/L increase in TT3, there is a potential 13 cm^2^ increase in visceral fat area (31). A number of studies have indicated that the geometric characteristics of the body roundness index may be capable of capturing the “metabolic overflow effect” that is precipitated by visceral fat accumulation. Once fat storage reaches its limit, the factors and inflammatory mediators secreted by fat cells undergo a change (32).

The present study identified a non-linear relationship between BRI and thyroid hormones, which may be primarily governed by the inflammation-deiodinase pathway. Additional mechanisms, including energy metabolism regulation, insulin resistance, and gut microbiota, also contribute to this relationship. At lower levels of BRI, moderate accumulation of visceral fat can promote leptin secretion, activating the hypothalamic–pituitary-thyroid (HPT) axis. This, in turn, enhances the secretion of thyrotropin-releasing hormone (TRH) and thyrotropin hormone (TSH), which in turn increases the activity of type II deiodinase (D2) and promotes the conversion of thyroxine (T4) to triiodothyronine (T3), resulting in elevated free triiodothyronine (FT3) levels (33, 34). The findings of this study suggest a correlation between the decrease in BRI and the subsequent increase in FT3, which aligns with the previously mentioned compensatory regulatory mechanisms. Furthermore, obesity is frequently linked to insulin resistance, and insulin has been observed to stimulate the conversion of T4 to T3. Consequently, a decline in insulin sensitivity may result in a further increase in T3 levels (35). The observed rise in TT3 within the lower BRI range also supports this mechanism. Once BRI exceeds a certain threshold, excessive accumulation of visceral fat triggers chronic low-grade inflammation, leading adipose tissue to continuously secrete inflammatory factors such as TNF-*α*, IL-6, and IFN-*γ*. These factors have been shown to directly inhibit the activity of thyroid follicular cells and peripheral tissue D2, weakening the conversion of T4 to T3 and causing the increase in FT3 to plateau or even decline (36–38). The results of this study demonstrate that when BRI > 7.21, the increase in FT3 diminishes or even reverses, which corroborates the dominant role of the inflammation-deiodinase mechanism. Furthermore, under inflammatory conditions, peripheral tissue sensitivity to thyroid hormones decreases, prompting the pituitary gland to secrete more TSH to maintain hormone homeostasis (39, 40). The present study found that TSH levels were significantly elevated in the high BRI group, which is consistent with the aforementioned mechanism. Furthermore, the expansion of visceral fat is associated with the upregulation of deiodinase inhibitors such as selenoproteins, which further limits thyroid hormone metabolism (41). It is important to note that dysbiosis of the gut microbiota has the potential to impair the gut barrier and increase the entry of inflammatory mediators such as lipopolysaccharides (LPS) into the bloodstream. This, in turn, can exacerbate systemic inflammatory responses and indirectly affect the synthesis and metabolism of thyroid hormones (42). Furthermore, the gut microbiome has been demonstrated to influence the absorption of trace elements, including iodine, selenium, zinc, and iron (43). For instance, zinc is a component of type 1 5′-deiodinase and participates in the conversion of T4 to T3 (44). The negative correlation observed between FT4 and BRI in our study suggests the potential involvement of gut and nutrition-related mechanisms.

In the context of this study, the alterations in TT3 and TT4 are influenced by a variety of mechanisms. According to extant research, serum total T3 levels are frequently elevated in cases of obesity. This phenomenon may be indicative of an augmented capacity of peripheral tissues to convert T4 to T3 in response to the metabolic demands of energy surplus (9). The findings of our study indicated that when BRI levels are within the lower to moderate range, there is an increase in TT3 with rising BRI. This observation suggests the presence of a metabolic compensation mechanism. However, as visceral fat further increases, chronic inflammatory factors such as TNF-*α* and adipose-derived signaling molecules gradually interfere with the secretion and synthesis of thyroid hormones, which may lead to decreased TT4 levels. Literature indicates that TNF-α can inhibit the expression of the sodium/iodine symporter (NIS) gene, reducing the uptake of iodine by thyroid follicular cells, thereby affecting hormone synthesis (36). The findings of this study demonstrate that an augmentation in BRI is concomitant with a diminution in TT4, thereby corroborating the inflammation-mediated mechanisms. Furthermore, the accumulation of visceral fat has been shown to regulate serum TT3 and TT4 levels indirectly through its influence on the concentration of thyroid hormone-binding proteins (32, 45).

In the subgroup analysis, a positive correlation was identified between BRI and FT3, TT3, and TT4, which was more pronounced among women, individuals with high dietary intake, and those with a university education. These findings imply that the sensitivity of thyroid hormones to visceral fat may be subject to variation based on gender and socioeconomic status. These disparities may be attributable to variations in sex hormones, nutritional status, and lifestyle factors. Gender differences may be associated with variations in responses to fat accumulation and hormone metabolism among women. Higher education levels and dietary intake have been linked to enhanced health awareness. Research has demonstrated a close relationship between mineral intake and thyroid dysfunction, underscoring the independent impact of dietary nutrition on thyroid health (19). The present study provides further evidence that populations with higher dietary antioxidant capacity are less likely to suffer from hypothyroidism, thereby supporting the hypothesis that dietary factors play a role in thyroid function. The negative correlation between FT4 and BRI manifested with greater clarity among male subjects and Mexican American populations; however, the statistical interaction did not attain statistical significance, a possibility attributable to inadequate sample size. The levels of thyroid-stimulating hormone (TSH) exhibited consistent variation across the study’s subgroups. This finding suggests that the regulatory mechanism of TSH remains relatively stable across diverse populations. The results underscore the necessity for a customized interpretation approach in clinical applications. This approach should take into account the unique characteristics of different populations to prevent the oversimplification of results. Furthermore, Chen et al. found a negative correlation between BRI and the risk of constipation, indicating that abdominal fat accumulation may indirectly affect thyroid hormone metabolism by regulating gut function (21).

BRI is anticipated to serve as a discerning metric in the assessment of the influence of visceral adipose tissue on thyroid function. This parameter may facilitate the timely identification and management of obesity-related thyroid dysfunction. Research indicates the presence of a substantial non-linear association and threshold effect between BRI and thyroid hormone levels, suggesting that regular monitoring of BRI can facilitate the early detection of mild changes in thyroid function among high-risk individuals, allowing for timely intervention (46). Given the importance of abnormal thyroid hormone levels as risk factors for various cardiovascular and metabolic diseases (39), a deeper understanding of the mechanisms through which visceral fat regulates thyroid function will contribute to the development of more scientifically sound prevention and treatment strategies. Furthermore, delineating the metabolic threshold of BRI will facilitate the identification of high-risk populations and enhance individualized management strategies (47). It is important to note that this study is not without its limitations. Firstly, as a cross-sectional study, it is unable to establish causal relationships and can only reveal correlations between BRI and thyroid hormones. Secondly, some covariates were missing. Despite the utilization of contemporary statistical methodologies to address this, potential biases may persist. Thirdly, the diagnosis of thyroid disease was based on disease coding, lacking specific clinical information, which limits the assessment of disease severity. Fourthly, the study population was predominantly composed of individuals from the United States, which may limit the generalizability of the findings to other ethnic groups or age ranges. A paucity of data regarding intervention measures was identified as a potential contributor to the underestimation of the true association between obesity and thyroid function.

Future research is recommended to conduct longitudinal cohort studies to verify causal pathways and use molecular biology techniques to explore the specific mechanisms of BRI in regulating thyroid hormones, particularly the role of adipose tissue-derived factors and inflammatory mediators. Furthermore, endeavors must be undertaken to broaden the demographic composition of the study population, thereby enhancing the generalizability of the findings.

## 6 Conclusion

The analysis, which was based on the NHANES database, found a non-linear association between the visceral fat index BRI and thyroid hormone levels. This association indicates that different BRI values correspond to distinct thyroid hormone states. This finding underscores the importance of visceral fat distribution characteristics in the assessment of thyroid function. Future research should further explore the potential mechanisms underlying the relationship between BRI and thyroid hormones, particularly the role of adipose tissue-derived factors and inflammatory mediators.

## Data Availability

The original contributions presented in the study are included in the article/Supplementary material, further inquiries can be directed to the corresponding author.
