# Supplementary material for: Body roundness index, thyroid hormones, and threshold effects in US adults: a cross-sectional study from NHANES
Source: Front Nutr. 2025 Jul 3;12:1539022. doi: 10.3389/fnut.2025.1539022 (PMC12267043; doi:10.3389/fnut.2025.1539022)
Supplement: Supplementary file 1 [file Table_1.docx]

**Supplementary Material**

**Supplemental Material S1**

**Consistency Analysis of Missing Data and Dummy Variable Treatment Results**

In this study, we addressed the missing data across several covariates using the dummy variable method to ensure data completeness and the robustness of our analyses. Table S1 presents the results of the association analysis between Body Roundness Index (BRI) and thyroid hormones (FT3, TT3, FT4, TT4, and TSH), which include results after handling the missing data. The following is a detailed description of the consistency between the missing data and the outcomes.

**1. Overview of Missing Data**

Table S1 outlines the missing data for each covariate, with overall missing rates being relatively low and deemed acceptable. For instance, the missing rate for the Poverty Income Ratio (PIR) is 8.47%, while the missing rate for special diet is 3.41%. Although missing data was present, the vast majority of covariates had missing rates below 15%, which justified the application of our data handling approach.

**2. Dummy Variable Treatment Method**

To mitigate bias arising from missing values, we employed the dummy variable method for the covariates with missing data. This approach involved creating an indicator variable (i.e., a dummy variable) for each covariate with missing values to signify the presence of missing data, thus ensuring that all valid information was retained in the analysis model.

**3. Consistency and Result Comparison**

The results obtained using the dummy variable method demonstrated good consistency with the original data analysis across different models. For example, in the analysis of FT3 and TT3, the regression coefficient for BRI (continuous) in Model 3 was 0.01 (95% CI: 0.00, 0.01). Whether using the complete dataset or the cleaned dataset, the results showed no significant differences and maintained the same trend.

**4. Statistical Significance**

The results following the treatment showed statistical significance comparable to those from the original data. For instance, the P-value indicated a strong positive correlation between BRI and TT3 in both datasets (P < 0.01), further supporting our conclusions on the association between BRI and thyroid hormones.

**5. Summary**

Overall, the relationships between the covariates and thyroid hormone levels remained consistent following the handling of missing data through dummy variables. This indicates that our method for addressing missing data is effective and that the data analysis results are reliable. Therefore, we can conclude that the association between BRI and thyroid hormones is unaffected by missing data, thereby enhancing the robustness of our study findings.

**Table S1.** Association Between BRI and Thyroid Hormones in Two Datasets

|  | **Model 3 [β (95% CI)]**  **N=7874** | **Model 3 [β (95% CI)]**  **N=7278(missing data)** |
| --- | --- | --- |
| FT3（pg/ml） |  |  |
| BRI (continuous) | 0.01 (0.00, 0.01) | 0.01 (0.00, 0.01) |
| BRI(quartile) |  |  |
| Quartile1 | Ref. | Ref. |
| Quartile2 | 0.03 (-0.00, 0.07) | 0.02 (-0.01, 0.06) |
| Quartile3 | 0.03 (-0.01, 0.07) | 0.03 (-0.01, 0.07) |
| Quartile4 | 0.07 (0.03, 0.11) | 0.07 (0.03, 0.11) |
| P for trend | 0.02 (0.01, 0.03) | 0.02 (0.01, 0.03) |
| TT3(n/dl) |  |  |
| BRI (continuous) | 0.95 (0.68, 1.23) | 1.01 (0.73, 1.30) |
| BRI(quartile) |  |  |
| Quartile1 | Ref. | Ref. |
| Quartile2 | 3.22 (1.52, 4.93) | 2.83 (1.04, 4.61) |
| Quartile3 | 4.82 (3.02, 6.61) | 4.67 (2.79, 6.55) |
| Quartile4 | 7.65 (5.76, 9.54) | 7.69 (5.71, 9.66) |
| P for trend | 2.42 (1.83, 3.01) | 2.48 (1.87, 3.10) |
| FT4（ng/dl） |  |  |
| BRI (continuous) | -0.03 (-0.04, -0.01) | -0.02 (-0.04, -0.00) |
| BRI(quartile) |  |  |
| Quartile1 | Ref. | Ref. |
| Quartile2 | -0.09 (-0.21, 0.02) | -0.13 (-0.25, -0.00) |
| Quartile3 | -0.23 (-0.36, -0.11) | -0.27 (-0.40, -0.14) |
| Quartile4 | -0.19 (-0.33, -0.06) | -0.21 (-0.35, -0.07) |
| P for trend | -0.07 (-0.11, -0.03), | -0.07 (-0.11, -0.03) |
| TT4（ug/dl） |  |  |
| BRI (continuous) | 0.06 (0.04, 0.08) | 0.06 (0.04, 0.08) |
| BRI(quartile) |  |  |
| Quartile1 | Ref. | Ref. |
| Quartile2 | 0.04 (-0.07, 0.16) | 0.01 (-0.11, 0.13) |
| Quartile3 | 0.14 (0.01, 0.26) | 0.11 (-0.01, 0.24) |
| Quartile4 | 0.26 (0.14, 0.39) | 0.23 (0.10, 0.37) |
| P for trend | 0.09 (0.05, 0.13) | 0.09 (0.05, 0.13) |
| TSH（miu/u） |  |  |
| BRI (continuous) | 0.03 (-0.00, 0.06) | 0.01 (-0.03, 0.04) |
| BRI(quartile) |  |  |
| Quartile1 | Ref. | Ref. |
| Quartile2 | -0.12 (-0.33, 0.08) | -0.09 (-0.30, 0.12) |
| Quartile3 | -0.15 (-0.37, 0.07) | -0.15 (-0.37, 0.08) |
| Quartile4 | -0.04 (-0.26, 0.19) | -0.05 (-0.28, 0.19) |
| P for trend | -0.00 (-0.07, 0.07) | -0.01 (-0.08, 0.06) |

^a^Model definition: Model 3: Adjusted for covariates after imputation and those with missing data, including age, sex, race, education level, hypertension, Poverty Income Ratio (PIR), diabetes, triglycerides (TG), thyroid peroxidase antibodies (TPOAB), special diet, total food intake (g), total cholesterol (mmol/L), and direct high-density lipoprotein cholesterol (Direct HDL-C).

^b^Trend test (P for trend): Based on the analysis of ordered variables of BRI quartiles.

^c^Abbreviations: BRI, Body Roundness Index; PIR, Poverty Income Ratio; FT3, free triiodothyronine; TT3, total triiodothyronine; FT4, free thyroid hormone; TT4, total thyroid hormone; TSH, thyroid-stimulating hormone.

^d^ Statistical significance markings: P < 0.05, *P < 0.01, **P < 0.001.
